# Multiple Somatic Mutations of SMARCA4 in Small Cell Carcinoma of the Ovary, Hypercalcemic Type: A Case Report

**DOI:** 10.7759/cureus.60802

**Published:** 2024-05-21

**Authors:** Yoko Aoyagi, Kentaro Kai, Haruto Nishida, Saki Aso, Eiji Kobayashi

**Affiliations:** 1 Obstetrics and Gynecology, Faculty of Medicine, Oita University, Yufu, JPN; 2 Diagnostic Pathology, Faculty of Medicine, Oita University, Yufu, JPN

**Keywords:** multiple mutations, smarca4, hypercalcemia, ovarian neoplasm, small cell carcinoma

## Abstract

Small-cell carcinoma of the ovary, the hypercalcemic type (SCCOHT) is a rare, aggressive tumor that primarily affects young females. It is a monogenic disorder caused by germline and/or somatic *SWI/SNF-related, matrix-associated, actin-dependent regulator of chromatin, subfamily a, member 4 (SMARCA4) *mutations. Here, we report a case of SCCOHT harboring multiple previously unreported somatic mutations in *SMARCA4* (c.2866_2867delC>T; c.3543del). A 28-year-old breastfeeding Japanese female presented to a previous hospital with nausea and vomiting. She had no family history of relevant malignancies, including ovarian cancer. Based on an evaluation performed at another institution, she was referred to a gynecologist for suspected ovarian cancer. Imaging studies revealed a 16×15 cm heterogenous enhancing mass within the right ovary without lymph node or distant metastasis. She had mild ascites without peritoneal dissemination, but there was an elevation in the serum calcium level (15.1 mg/dL). The patient underwent cytoreductive surgery and was pathologically diagnosed with SCCOHT. Auxiliary immunohistochemical staining confirmed the loss of SMARCA4 protein expression. The patient was diagnosed with the International Federation of Gynecology and Obstetrics (FIGO) 2014 stage IA (pT1a pN0 M0). The serum calcium levels returned to normal post-surgery. Matched-pair analysis using tumor tissue and peripheral blood revealed multiple somatic mutations in *SMARCA4*, but no deleterious germline mutations were present. Microsatellite instability was not significant, and the patients had a heterozygous mutation of uridine diphosphate glucuronosyl transferase 1A1 (UGT1A1)*6. She underwent six cycles of irinotecan hydrochloride plus cisplatin chemotherapy and achieved complete remission. The patient was finally examined and evaluated 45 months postoperatively; there was no evidence of the disease. Overall, the genetic findings will not aid in the SCCOHT diagnosis and relevant genetic counseling; however, they may have implications for the treatment of this disease in the future.

## Introduction

Switch/sucrose non-fermentable (SWI/SNF) chromatin complexes play an important role in gene transcription, cell fate control, and lineage specification [1]. *SWI/SNF-related, matrix-associated, actin-dependent regulator of chromatin, subfamily a, member 4 (SMARCA4)* is one of the most frequently mutated chromatin-remodeling ATPases in the SWI/SNF complex and acts as a tumor suppressor gene in cancer [2].* SMARCA4* germline mutations are evident in almost all infant rhabdoid tumor predisposition syndrome-2 (RTPS2) and congenital multiple malformation syndrome (Coffin-Siris syndrome), approximately 35% of rhabdoid tumors, and approximately 50% of small cell carcinoma of the ovary, hypercalcemic type (SCCOHT) [3]. *SMARCA4* somatic mutations have been detected at variable frequencies in gastrointestinal, pulmonary, and uterine malignancies [3].

SCCOHT, which occurs in 0.12 per one million persons per year [4], has an aggressive clinical picture with 50% two-year survival; it primarily affects young females in their 20s [5]. In more than 60% of cases, SCCOHT manifests with hypercalcemia and can present as a paraneoplastic syndrome [5]. SCCOHT was first identified in 2014 as a monogenic disorder caused by germline and/or somatic *SMARCA4* mutations [6-8]. Owing to its rare incidence, clinicians face challenges associated with its diagnosis, genetic counseling, and treatment although some clinical management guidelines have been published [9,10].

Here, we report an unexpected case of SCCOHT that was identified by rapid intraoperative diagnosis and a successful postoperative treatment course with platinum-based chemotherapy. In addition, we reviewed the previously reported patterns of multiple somatic mutations in *SMARCA4*.

## Case presentation

**Figure 1:**
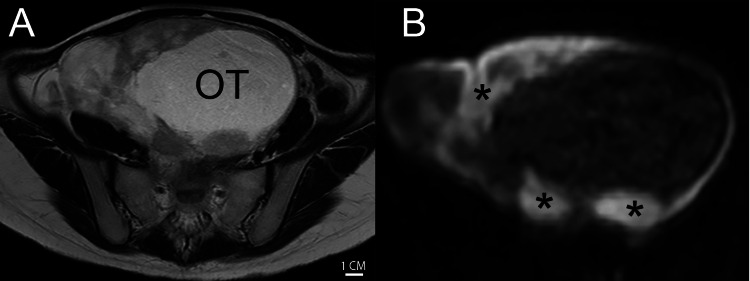
Preoperative MRI of the pelvis. (A) T2-weighted axial view, demonstrating a 16×15 cm mixed cystic-solid pattern mass with a dominant cystic part. OT, ovarian tumor. Scale bar, 1 cm. (B) Diffusion-weighted axial view, demonstrating diffusion restriction in peripheral solid parts (*).

A 28-year-old gravida 1, para 1, breastfeeding Japanese female presented with nausea and vomiting. Based on the transvaginal evaluation performed by a primary OB-GYN care physician, the patient was referred to a previous hospital for a suspected ovarian tumor. At the time of presentation, the patient reported an increase in the pelvic pressure. The serum carbohydrate antigen 125 (CA125), CA19-9, alpha-fetoprotein (AFP), and soluble interleukin-2 receptor (sIL-2R) levels were normal. However, the serum calcium level was elevated (15.1 mg/dL; reference range: 8.8-10.1 mg/dL), and the electrocardiogram showed QT shortening. The patient had no remarkable medical, surgical, allergic, and social histories. She had no regular medication and no family history of relevant malignancies, including ovarian cancer. She was married and gave birth eight months earlier. She did not smoke, drink alcohol, or consume illicit drugs. Pelvic and speculum examinations were not significant, but bimanual examination confirmed a pelvic tumor, the size of a newborn baby’s head, extending to the navel. Contrast-enhanced magnetic resonance imaging (MRI) revealed a 16×15 cm heterogenous enhancing mass in the right ovary (Figure 1). A computed tomography scan (CT) revealed no lymph node involvement, peritoneal dissemination, or distant metastases. The peritoneal ascites were mild and not clinically significant. According to the latest classification system at that time, we tentatively diagnosed the patient with the International Federation of Gynecology and Obstetrics (FIGO) 2014 stage IA ovarian cancer (cT1a cN0 M0).

**Figure 2:**
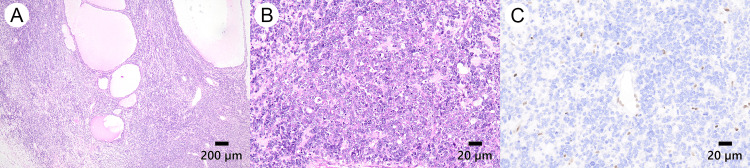
Pathological assessment of the resected specimen. (A) Hematoxylin and eosin stain show diffuse tumor cell infiltration. Focally, it demonstrates follicle-like structures. Original magnification 4×. Scale bar, 200 μm. (B) Hematoxylin and eosin stain, demonstrating large nests of small cells like small-cell lung cancer with fibrous stroma. Original magnification 40×. Scale bar, 20 μm. (c) Immunohistochemical stain demonstrating loss of immunoreactivity for SMARCA4 in the tumor cell; positive for lymphocyte and vascular endothelial cells as a control. Original magnification 40×. Scale bar, 20 μm.

The patient originally planned to undergo fertility-sparing surgery but abandoned it because the intraoperative rapid diagnosis revealed SCCOHT. We performed a cytoreductive surgery with no residual disease consisting of bilateral oophorectomy, extra-fascial hysterectomy, pelvic and para-aortic lymph node dissection, and partial omentectomy. The operation duration was 5 h 24 min, and the estimated blood loss was 509 mL. The postoperative course of the patient was uneventful, and her serum calcium levels returned to normal on postoperative day one. Peritoneal washing revealed no malignancy. Microscopically, the resected specimen revealed the presence of a malignant tumor composed of irregular diffuse proliferation of relatively small, atypical cells (Figures 2A, 2B). Immunohistochemically, the tumor cells were partially positive for cytokeratin (AE1/AE3), epithelial membrane antigen (EMA), cluster of differentiate 56 (CD56), synaptophysin, and CD10 and loss for *SMARCA4 *(Figure 2C), chromogranin A, and thyroid transcription factor-1 (TTF-1). The patient was diagnosed with FIGO stage IA SCCOHT (pTa1 pN0 M0) and was referred to our department for postoperative adjuvant therapy and genomic evaluation.

Cancer-related genetic mutations were screened for after conducting deep genetic counseling with her husband and mother. Gene panel testing using the OncoGuideTM National Cancer Center (NCC) Oncopanel System (National Cancer Center, Tokyo, Japan, and Sysmex Corporation, Kobe, Japan) revealed two pathogenic somatic mutations of *SMARCA4* in the tumor (c.2866_2867delC>T [p.L956fs*2] and c.3543delC [p.Q1182fs*34]). There was 7,366 bp between the two distinct mutations, and the sequence read length (~150 bp) was too short to identify whether these mutations were in the same allele (*cis-*) or not (*trans-*). No pathogenic germline mutations were evident by performing matched-pair analysis (tumor-normal pair) using peripheral blood and tumor specimens. She had another germline nonsynonymous single-nucleotide variant in the *TSC complex subunit 1 (TSC1)*, which was judged as benign/likely benign according to the ClinVar database (https://www.ncbi.nlm.nih.gov/clinvar/). The tumor mutation burden was low at 5.6/Mb, and microsatellite instability was not significant. The tumor cell content in the specimens used in this assay was 80%. The variant allele frequency was 43.6% for c.2866_2867delC>T and 45.0% for c.3543delC, which was consistent with heterozygous alterations. Analysis of polymorphisms in *uridine diphosphate glucuronosyl transferase 1A1 (UGT1A1)* for irinotecan showed that the patients had a heterozygous mutation of UGT1A1*6. The patient was administered systemic chemotherapy with irinotecan hydrochloride (60 mg/m^2^, IV, days one, eight, and 15) and cisplatin (60 mg/m^2^, IV, day one) every 28 days. The patient did not experience any adverse effects greater than grade 3/4 based on the Common Terminology Criteria for Adverse Events (CTCAE, v4.0). However, she underwent lymphatic venous shunt placement for grade 1 lymphedema in the left lower limb three times, and she was administered estrogen replacement therapy for a climacteric disorder. The patient was finally examined 45 months postoperatively; there was no evidence of the disease.

All the aforementioned medical procedures were provided by the health insurance system in Japan. Due to the retrospective nature of this case report, the need for institutional review board approval was waived. Written informed consent was obtained from the patient and her husband for the publication of anonymized information in this article.

## Discussion

This case report describes two important clinical findings. First, we encountered a rare case of SCCOHT harboring multiple previously unreported somatic mutations in *SMARCA4*. Second, the patient underwent cytoreductive surgery, followed by the administration of platinum-based combination chemotherapy, and remained progression-free for 45 months.

**Figure 3:**
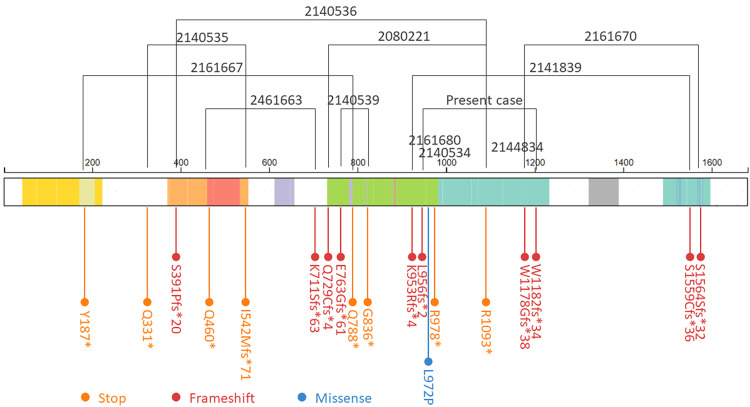
Distribution of multiple mutations in SMARCA4. The data were collected from genome-wide studies annotated in the COSMIC database (release version 99).

In our case report, there were two non-synonymous somatic mutations in the *SMARCA4* gene. To date, we examined 54 *SMARCA4* mutation patterns in 41 SCCOHT cases from the Catalog of Somatic Mutations in Cancer (COSMIC) database [11]. Among them, 28 (68.3%) cases had a single mutation, and 13 (31.7%) had distinct multiple mutations (MMs) in *SMARCA4*. MMs [12], which are also known as composite mutations [13], are defined as two or more distinct somatic mutations in the same gene. Additionally, MMs are subdivided into cis- (two mutations in the same allele) and trans- (two mutations in different alleles); approximately 75% of the MMs in tumor suppressor genes, such as *SMARCA4*, are of the trans-type [13]. We demonstrated and listed a total of 13 MMs that were previously reported cases in COSMIC (11 cases, excluding two cases in which the amino acid mutation pattern was not determined) and our case in Figure 3 and Table 1 [6-8,14], which showed that the MMs in *SMARCA4* were distributed throughout their length. This pattern was concordant with that observed in other tumor suppressor genes [15]. However, this type of MM is strongly biased toward truncating mutations (non-sense/stop and frameshift). This pattern was not in concordance with the pan-cancer analysis of 5,954 mutations in which the missense mutations accounted for over 70% of all mutations [13]. Collectively, we speculated that the loss of function via trans biallelic mutations in *SMARCA4* led to tumor development in this case.

**Table 1:** SMARCA4 multiple somatic mutations in SCCOHT. AA, amino acids; Bromo_SNF2L2, Bromodomain, SNF2L2-like subfamily; CDS, coding sequence; DEXDc, DEXH-box helicase domain; COSMIC, Catalog of Somatic Mutations in Cancer; HELICc, helicase c; QLQ, pfam08880; SNF2_N, SNF2 family N-terminal domain

#	COSMIC sample ID	AA Mutation	CDS Mutation	Zygosity	Mutation type	Functional domain	References
1	2080221	p.Q729Cfs*4	c.2184_2206del	Unknown	Frameshift	Nonfunctional	Kupryjańczyk et al. [14]
		p.R1093*	c.3277C>T	Unknown	Stop	HELICc	
2	2140534	Unknown	c.1761+1G>A	Heterozygous	Splice	Nonfunctional	Jelinic et al. [8]
		p.R978*	c.2932C>T	Heterozygous	Stop	SNF2_N	
3	2140535	p.Q331*	c.991C>T	Heterozygous	Stop	Nonfunctional	Jelinic et al. [8]
		p.I542Mfs*71	c.1626del	Heterozygous	Splice	Bromo_SNF2L2	
4	2140536	p.S391Pfs*20	c.1167del	Heterozygous	Frameshift	Nonfunctional	Jelinic et al. [8]
		p.R1093*	c.3277C>T	Heterozygous	Stop	HELICc	
5	2140539	p.E763Gfs*61	c.2287dup	Heterozygous	Frameshift	Nonfunctional	Jelinic et al. [8]
		p.G836*	c.2506G>T	Heterozygous	Stop	DEXDc	
6	2141834	Unknown	c.2001+1del	Unknown	Frameshift	Unknown	Ramos et al. [7]
		p.L1161Sfs*3	c.3481del	Unknown	Frameshift	HELICc	
7	2141839	p.K953Rfs*4	c.2858del	Unknown	Frameshift	SNF2_N	Ramos et al. [7]
		p.S1559Cfs*36	c.4675_4678del	Unknown	Frameshift	Bromo_SNF2L2	
8	2161663	p.Q460*	c.1378C>T	Unknown	Stop	Nonfunctional	Witkowski et al. [6]
		p.K711Sfs*63	c.2130del	Unknown	Frameshift	Nonfunctional	
9	2161667	p.Y187*	c.561C>G	Unknown	Stop	QLQ	Witkowski et al. [6]
		p.Q788*	c.2362C>T	Unknown	Stop	DEXDc	
10	2161670	p.W1178Gfs*38	c.3531del	Heterozygous	Frameshift	HELICc	Witkowski et al. [6]
		p.I1564Sfs*32	c.4690del	Heterozygous	Frameshift	Bromo_SNF2L2	
11	2161680	p.L972P	c.2915T>C	Heterozygous	Missense	SNF2_N	Witkowski et al. [6]
		Unknown	c.3168+1G>C	Heterozygous	Splice	Unknown	
12	Present case	L956fs*2	c.2866_2867delC>T	Heterozygous	Frameshift	SNF2_N	Present study
		Q1182fs*34	c.3543delC	Heterozygous	Frameshift	HELICc	

Cytoreductive surgery, followed by the administration of platinum-based chemotherapy, led to complete remission, which lasted for 45 months. In the largest case series of 293 SCCOHTs, the stage at diagnosis in all patients and treatment modality (adjuvant chemotherapy and/or radiotherapy and high-dose chemotherapy with autologous stem cell rescue) in patients with stages II-IV were independent prognostic factors [16]. A systematic literature review of 135 SCCOHT cases reported that the most commonly used regimens were cisplatin, carboplatin, and etoposide [17]. This could be attributed to several reasons. First, the tumor was misinterpreted by some investigators as a small cell carcinoma of the ovary, pulmonary type (SCCOPT) or neuroendocrine type. Historically, the term “hypercalcemic type” was later added to “small cell carcinoma” to distinguish between SCCOPT and SCCOHT [18]. Second, the tumor showed variable expression of neuroendocrine markers by immunohistochemistry. A nationwide study on ovarian neuroendocrine neoplasms (NENs) in Japan collected 68 NEN patients from 20 institutions, but three of them were excluded due to SCCOHT [19]. Finally, an appropriate histology-specific regimen has yet to be established. SCCOHT was recategorized into miscellaneous tumors according to the World Health Organization classification revision in 2020, yet exploration of findings of the epithelial ovarian tumor to SCCOHT is a reasonable choice in a real-world setting.

Based on the literature review, there was no considerable distinction in the choice of treatment for stage I SCCOHT (cytoreductive surgery, followed by platinum-based chemotherapy). Therefore, the patient’s stage at diagnosis (stage I) may have contributed to her long-term survival. Witkowski et al. [6] compared survival in patients with SCCOHT according to the mutation origin (somatic or germline) and found that patients with somatic mutations showed better, but non-significant, survival rates compared to those with germline mutations. We extrapolated the available evidence on small-cell lung carcinoma (Irinotecan plus cisplatin) to the present case of SCCOHT, as it was pathologically characterized by adenocarcinoma with partly neuroendocrine differentiation. The 2020 International SCCOHT Consortium recommends the off-label use of immune-checkpoint blockade agents if all the other treatment options fail. A strong association between T-cell infiltration and programmed cell death ligand (PD-L1) expression was reported in 10 of 11 SCCOHT cases with a low mutation burden [20].

## Conclusions

Here, we report a case of SCCOHT with novel somatic MMs in *SMARCA4*; the patient exhibited long-term survival without disease after undergoing cytoreductive surgery and the administration of platinum-based systemic chemotherapy. The clinical implications and roles of MMs, such as drug sensitivity, in the prognosis of SCCOHT remain unknown. Therefore, further studies are warranted to compare the clinical outcomes between different types of mutations (single or cis- and trans-). Both clinicopathological and genomic approaches are warranted to decipher the underlying mechanism of the aggressive behavior of SCCOHT owing to its low prevalence.
